# Impact of Pre‐Treatment Comorbidity Burden on Survival in Patients Receiving Venetoclax Plus Hypomethylating Agents

**DOI:** 10.1002/ajh.27591

**Published:** 2025-01-20

**Authors:** Giovanni Marconi, Elisabetta Petracci, Giuseppe Lanzarone, Calogero Vetro, Maria Paola Martelli, Cristina Papayannidis, Ernesta Audisio, Paola Minetto, Carola Riva, Fabio Guolo, Gianluca Martini, Patrizia Zappasodi, Federico Vincenzo, Federica Gigli, Davide Griguolo, Michela Rondoni, Giulia Ciotti, Erika Borlenghi, Nadia Ciccone, Fanny Erika Palumbo, Jacopo Nanni, Daniele Mattei, Massimo Bernardi, Alessandro Cignetti, Chiara Zingaretti, Bernadette Vertogen, Claudio Cerchione, Francesco Lanza, Daniela Cilloni, Lorenzo Brunetti, Raffaele Palmieri, Antonio Curti, Maria Benedetta Giannini, Elisabetta Todisco, AVALON research group, Nicola Fracchiolla, Giovanni Martinelli

**Affiliations:** ^1^ IRCCS Istituto Romagnolo per lo Studio dei Tumori (IRST) “Dino Amadori” Meldola Italy; ^2^ Ematologia U, AOU Città della Salute e della Scienza di Torino Torino Italy; ^3^ Divisione di Ematologia AOU Policlinico “G. Rodolico‐San Marco” Catania Italy; ^4^ UOC Ematologia, Azienda Sanitaria dell'Alto Adige Bolzano Italy; ^5^ Dipartimento di Medicina e Chirurgia Università di Perugia, Ospedale “Santa Maria della Misericordia” Perugia Italy; ^6^ IRCCS Azienda Ospedaliero‐Universitaria di Bologna, Istituto di Ematologia “Seràgnoli” Bologna Italy; ^7^ Complex Structure of Hematology, AO Città della Salute e della Scienza Torino Italy; ^8^ Clinic of Hematology, Department of Internal Medicine University of Genova Genova Italy; ^9^ Department of Molecular Medicine University of Pavia Pavia Italy; ^10^ Dipartimento di Oncoematologia Fondazione IRCCS Policlinico “San Matteo” Pavia Italy; ^11^ Unità di Ematologia e TCS, Ospedale “Vito Fazzi” Lecce Italy; ^12^ Divisione di Oncoematologia IRCCS Istituto Europeo di Oncologia Milano Italy; ^13^ SC Ematologia, ASU Giuliano Isontina Trieste Italy; ^14^ UO Ematologia, Ospedale S. Maria delle Croci, AUSL Romagna Ravenna Italy; ^15^ Onco Hematology, Department of Oncology Veneto Institute of Oncology IOV‐IRCCS Padua Italy; ^16^ Spedali Civili, Hematology Brescia Kentucky USA; ^17^ S.C. Ematologia, Azienda Ospedaliero Universitaria di Ferrara Ferrara Italy; ^18^ Complex Structure of Hematology, ASO S. Croce e Carle Cuneo Italy; ^19^ UO Ematologia e Centro Trapianto di Midollo Osseo, IRCCS Ospedale “San Raffaele” Milano Italy; ^20^ SCDU di Ematologia e Terapie Cellulari, AO Ordine Mauriziano Torino Italy; ^21^ Department of Clinical and Biological Sciences University of Turin Orbassano Italy; ^22^ Department of Clinical and Molecular Sciences Università Politecnica delle Marche Ancona Italy; ^23^ Department of Biomedicine and Prevention University of Rome Tor Vergata Rome Italy; ^24^ SC Ematologia, Ospedale “Busto Arsizio”, ASST Valle Olona Varese Italy; ^25^ UOC Oncoematologia, Fondazione IRCCS “Ca'Granda” Ospedale Maggiore Policlinico Milano Italy

**Keywords:** acute myeloid leukemia, AML, fitness, low intensity therapy

## Abstract

Expectation of survival of patients receiving HMA + VEN is influenced by pre‐treatment comorbidity burden.

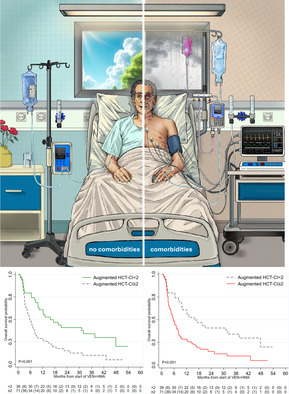

To the Editor,

Acute Myeloid Leukemia (AML) presents significant treatment challenges, especially in patients unfit for intensive chemotherapy due to age or clinical condition. Venetoclax (VEN) in combination with hypomethylating agents (HMA) has shown promise in improving outcomes in these populations. In the AVALON study, we previously reported valuable evidence into this combination's real‐world effectiveness and safety [1]. SIE/SIES/GITMO consensus criteria was developed to assess feasibility of intensive chemotherapy [2], however, it demonstrated to be discriminative for prognosis of newly diagnosed patients who receive VEN + HMA, especially in the “frail” population, where SIE/SIES/GITMO criteria retain prognostic capabilities with low‐intensity therapy [3]. Recently, new risk classifications based on molecular biology were also proposed [4]. However, albeit these criteria can identify patients with poor outcomes after VEN + HMA therapy, they fall short in defining frailties that can be managed and consequently improved with medical interventions [3, 5]. We recently reported that the pre‐treatment comorbidity burden may affect survival in patients treated with single‐agent HMA independently of disease biology [6]. Consequently, we collected pre‐treatment comorbidities as defined by Sorror and colleagues [6, 7] in the AVALON patient set and tested their impact on prognosis. Here, we expand our findings emphasizing the need to understand how pre‐treatment clinical variables influence therapeutic outcomes.

AVALON is a multicenter, observational cohort study that enrolled 232 AML patients across 32 Italian centers within 2015 and 2020, of which 190 received VEN + HMA for the treatment of relapsed or refractory AML. After queries and data monitoring, a total of 143 patients were included in this sub‐analysis of pre‐treatment comorbidity burden (CONSORT diagram is presented in Supporting Information S1). The excluded patients had a superimposable outcome compared to the patients included in these analyses, as shown in Supporting Information S2. Analyzed data comprised patient demographics, treatment regimens, response rates, survival outcomes, and adverse events. The cohort included both newly diagnosed (*n* = 36, 25.2%) and relapsed/refractory (R/R, *n* = 107, 74.8%) AML patients. Overall response rate and survival data of the population included in the analysis echoed the current literature; the safety profile was consistent with previous reports about VEN + HMA regimen [1].

For the purposes of the analysis, we decided to evaluate fitness scores based on comorbidities, namely, HCT‐CI and augmented HCT‐CI. HCT‐CI is a comorbidity index originally developed for allogeneic transplant and validated in fit and unfit AML patients that incorporate relevant end‐organ damages and morbidities, augmented HCT‐CI also include albumin, platelet. and LDH levels [6, 7]. Importantly, the definition of pre‐treatment comorbidities was adherent with clinical practice, collected from available data and therefore was not based on pre‐planned standardized exams. This should be underlined for the interpretation of the results of this study and accounted as a limitation. Pre‐treatment comorbidities are presented in Supporting Information S3; a significant portion of patients had valvular disease (*n* = 4, 2.8%), arrhythmia (*n* = 5, 3.5%) or non‐valvular and non‐electric cardiovascular comorbidities (*n* = 20, 14.0%), lung diseases (*n* = 15, 10.5%), or prior malignancies (*n* = 17, 11.9%). Furthermore, thrombocytopenia (< 20 × 10^9^/L) was present in 83 patients (58.0%), hypoalbuminemia in 11 patients (7.7%), and elevated LDH in 64 patients (44.8%).

HCT‐CI and augmented HCT‐CI were calculated based on these comorbidities only in patients without missing data. The median value of HCT‐CI and augmented HCT‐CI were 1 and 2, respectively. Patients were divided into two groups based on the median value. Secondary AML was more common within patients with higher HCT‐CI (34% vs. 22%, *p* = 0.009) and augmented HCT‐CI (37% vs. 18%, *p* = 0.015). The SIE/SIES/GITMO criteria were associated with HCT‐CI (*p* = 0.010) but not with augmented HCT‐CI (*p* = 0.272). However, for both scores, about half of SIE/SIES/GITMO fit patients presented high HCT‐CI and augmented HCT‐CI scores (44.2% and 57.1%, respectively, Supporting Information S4), and about one‐third of SIE/SIES/GITMO unfit for intensive CT patients presented low HCT‐CI and augmented HCT‐CI scores (32.3% and 28.0%, Supporting Information S4). Discrepancies within SIE/SIES/GITMO criteria and HCT‐CI or augmented HCT‐CI scores were driven by comorbidities whenever a SIE/SIES/GITMO fit patient was assigned to a high HCT‐CI/augmented HCT‐CI score and by ages and ECOG whenever SIE/SIES/GITMO unfit or frail patient was assigned to a low HCT‐CI/augmented HCT‐CI score. Other characteristics were similar across groups (Supporting Information S5). We than focused on the impact of any pre‐treatment comorbidity on overall survival (OS), as reported in Supporting Information S6; cardiovascular disease (HR [95% CI], 1.82 [1.09–3.04], *p* = 0.022), moderate or severe hepatic dysfunction (5.17 [1.24–21.57], *p* = 0.024), hypoalbuminemia (2.08 [1.07–4.03] *p* = 0.031), and thrombocytopenia (2.11 [1.42–3.14], *p* < 0.001) resulted associated with OS in univariate analysis. Thereafter, we focused on the prognostic impact of HCT‐CI and augmented HCT‐CI. Overall response including complete remission with or without hematologic recovery was obtained in 57 patients (39.9%) across the entire set; response rate was numerically higher in patients with HCT‐CI < 1 (32/64 vs. 25/79, 50% vs. 32%, *p* = 0.071) and with augmented HCT‐CI < 2 (22/39 vs. 26/71, 56% vs. 37%, *p* = 0.092), without reaching statistically significant differences. Patients with low augmented HCT‐CI had a significantly longer survival compared to those with higher values of the score (*p* = 0.001, Figure 1), with median OS of 15.8 months [95% CI 9.7–32.0] and of 5.7 months [95% CI 4.0–7.7], respectively. Similarly, patients with lower HCT‐CI score survived longer than those with higher scores (*p* = 0.081); median OS were 10.2 months [95% CI 7.7–15.8] and 6.3 months [95% CI 4.4–8.2], respectively. Event‐free survival was consistent with OS data (Supporting Information S7). The prognostic impact of comorbidity scores was not influenced by age, ELN risk, and secondary AML status, as tested by multivariable Cox regression analysis (Supporting Information S8).

**FIGURE 1:**
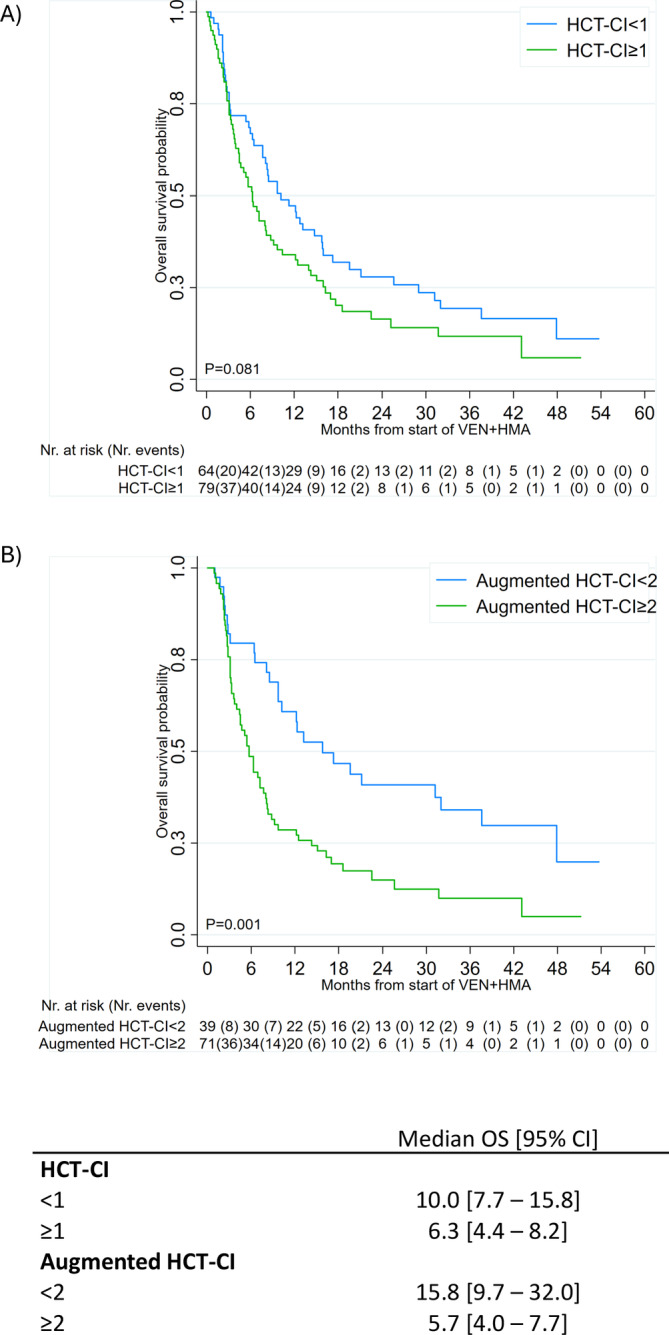
Kaplan–Meier curves for OS by HCT‐CI (A) and augmented HCT‐CI (B) classes based on the median value.

As far as we know, this is the first report of the utility of comorbidity scores in patients receiving VEN + HMAs. Overall, our data showed that patients with lower pre‐treatment comorbidities, especially as identified by low augmented HCT‐CI, had the best chance to benefit from VEN + AZA therapies. This was consistent also referring at score subgroups as defined by Sorror et al. [6] (Supporting Information S9), across young and elderly patients (Supporting Information S10 age < 60 or ≥ 60) and across newly diagnosed and R/R AML patients (Supporting Information S11). Thus, the impact of comorbidity score was independent on transplant eligibility or disease status. However, as expected, elderly and—consequently—newly diagnosed patients had higher comorbidity burden, and the population of elderly patients with low comorbidity scores was underrepresented in our set (Supporting Information S12).

Finally, we delve into demonstrating how pre‐treatment comorbidity defined prognosis. We speculated that a higher comorbidity burden would partially impact on patient's frailty, and then translate into a higher incidence of adverse events. We observed a higher exposure‐adjusted event rate (EAER) of non‐hematological grade ≥ 3 adverse events (AE) among patients with augmented HCT‐CI ≥ 2 compared to those having a lower burden (0.95 vs. 0.06). No difference was observed with respect to HCT‐CI, the EAER was of 0.10 in both groups defined on median cut‐off of 1. AML‐CM [6] was not used in the analysis since ELN2017‐based genetic risk included in this score is not well applicable to VEN + HMA treatment, as widely reported and demonstrated in our patient set in the multivariable regression model (Supporting Information S8). Regrettably, we were not able to evaluate consistently the impact of innovative molecular determinant of risk in this analysis, since receptor tyrosine kinases mutations and TP53 mutations were not homogeneously available in our set, and this is a limitation of our study [4].

Our findings contribute to the growing body of evidence supporting VEN + HMA efficacy and safety, while providing clinicians with critical information to guide treatment decisions. Pre‐treatment comorbidities represent the most important clinical aspect that can be the subject of active medical care, as it was also shown by the higher incidence of AEs in the categories with higher comorbidities. Herein, comorbidities screening is a pivot point before VEN + HMA treatment. Patients who are not candidates for active management of multiple comorbidities should be informed about the moderate benefit of VEN + HMA intervention; consequently, treatment indication in this population must be considered wisely. A careful evaluation of pre‐treatment and disease‐unrelated comorbidities seems to be an important aspect both to select overperforming patients that may be candidate to intensification after VEN + HMA (e.g., with transplant) and to select patients with dismal outcomes in which experiment different treatment modalities (e.g., less dose‐dense VEN administration) together with treatment rescue interventions. Finally, comorbidity evaluation may also be informative for a wise adjustment of VEN exposure during therapy courses that respect patient condition and ongoing complications, since fewer day of VEN may retain effectiveness in term of remission of induction, and in a frail subset, the continued administration of the drug may be poorly tollerable [8, 9].

While observational studies have inherent limitations, the breadth of our data offers a comprehensive perspective on the real‐life application and outcomes of this treatment. Our findings support the continued use and further optimization of VEN + HMA, with the potential to significantly improve patient outcomes. Integration of our results with comprehensive geriatric assessment and other pre‐treatment screenings is warranted in prospective cohorts.

## Author Contributions

Giovanni Marconi, Elisabetta Petracci, Giuseppe Lanzarone, Fabio Guolo, Gianluca Martini, Giulia Ciotti, Jacopo Nanni, Chiara Zingaretti, Lorenzo Brunetti, Francesco Lanza, Daniela Cilloni, Raffaele Palmieri, Antonio Curti, Nicola Fracchiolla, and Giovanni Martinelli conceived the analysis and contributed to the definition of methods and endpoints. Elisabetta Petracci performed statistical analysis. Chiara Zingaretti and Bernadette Vertogen were involved in study database creation and revision. Calogero Vetro, Maria Paola Martelli, Cristina Papayannidis, Ernesta Audisio, Carola Riva, Patrizia Zappasodi, Federico Vincenzo, Federica Gigli, Davide Griguolo, Michela Rondoni, Erika Borlenghi, Nadia Ciccone, Daniele Mattei, Massimo Bernardi, Alessandro Cignetti, Claudio Cerchione, Francesco Lanza, Maria Benedetta Giannini, Elisabetta Todisco, Nicola Fracchiolla, and the AVALON research group enrolled patients in the study. All the authors, including any AVALON research group, contributed in writing and reviewing the manuscript.

## Ethics Statement

The AVALON study was approved by the Romagna Ethics Committee on April 10, 2019 (Prot. 3371/2019), and subsequently by the ethics committee of each participating institution. It was also conducted in accordance with the ethical standards in the 1964 Declaration of Helsinki.

## Consent

Written informed consent from patients was not required because of the retrospective nature of the study.

## Conflicts of Interest

GMarc received research funds from AbbVie, Astellas, AstraZeneca, Daiichi Sankyo, Pfizer, and Syros and was a consultant or was included in the speaker's bureau for AbbVie, Astellas, AstraZeneca, Immunogen, Janssen, Menarini/Stemline, Pfizer, Ryvu, Servier, Syros, and Takeda. FL received research funds from Pfizer and Alexion and is a consultant from Sobi, Roche, AbbVie, Amgen, and Novartis. MR was a consultant or was included in the speaker's bureau for Novartis, Gentili, Blueprint, and Jazz. GMartinelli is a consultant for AbbVie Inc., Celgene, Roche, Janssen, Astellas, Pfizer, and Incyte. The remaining authors declare that the research was conducted in the absence of any commercial or financial relationships that could be construed as a potential conflicts of interest.

## Permission to Reproduce Material From Other Sources

All the resources presented in this article can be reproduced.

## Supporting information


Data S1.

## Data Availability

Questions regarding data sharing should be addressed to the corresponding author, data will be shared upon request.
