# Supplementary material for: Impact of Pre‐Treatment Comorbidity Burden on Survival in Patients Receiving Venetoclax Plus Hypomethylating Agents
Source: Am J Hematol. 2025 Jan 20;100(4):708–11. doi: 10.1002/ajh.27591 (PMC11886493; doi:10.1002/ajh.27591)
Supplement: Supplementary file 1 — Data S1. [file AJH-100-708-s001.docx]

Supplementary

S1: consort diagram of patients considered for this analysis. HCT-CI: Hematopoietic cell transplantation comorbidity index.

232 enrolled patients

218 patients

14 patients were excluded for insufficient clinical data

190 patients

28 patients were excluded for other therapies:

- Venetoclax single agent (16)
- Venetoclax + intensive chemotherapy (4)
- Venetoclax + low dose cytarabine (8)

143 evaluable patients for HCT-CI

47 patients were excluded for inaccurate or missing pre-treatment comorbidities data

110 evaluable patients for augmented HCT-CI

33 patients were excluded only from augmented HCT-CI analyses for lack of pre-treatment LDH, platelet, or albumin

Primary analysis

S2: Kaplan-Meier curves for OS of patients excluded from this analysis for inaccurate collection of pre-treatment comorbidities vs patients evaluable for HCT-CI (A) or augmented HCT-CI (B), as per S1

A)


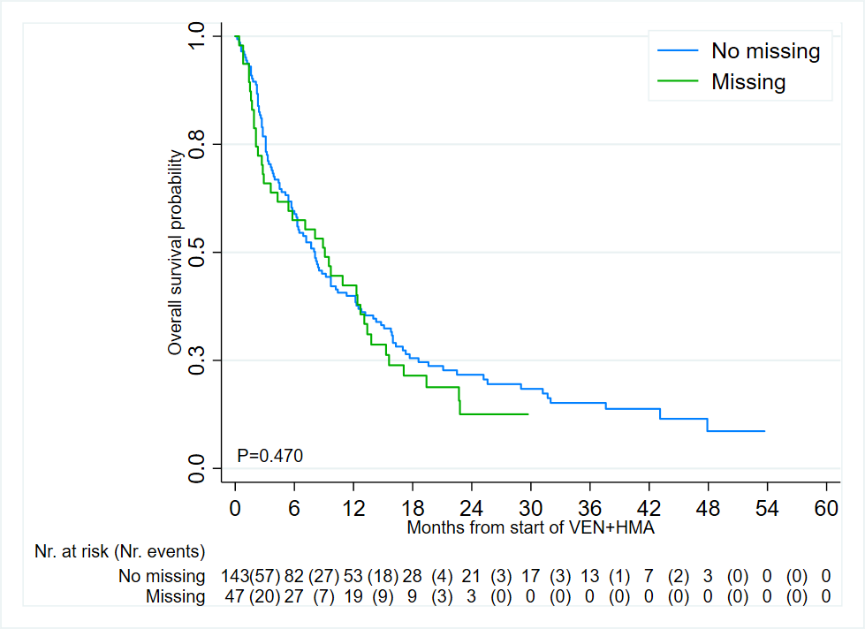


B)


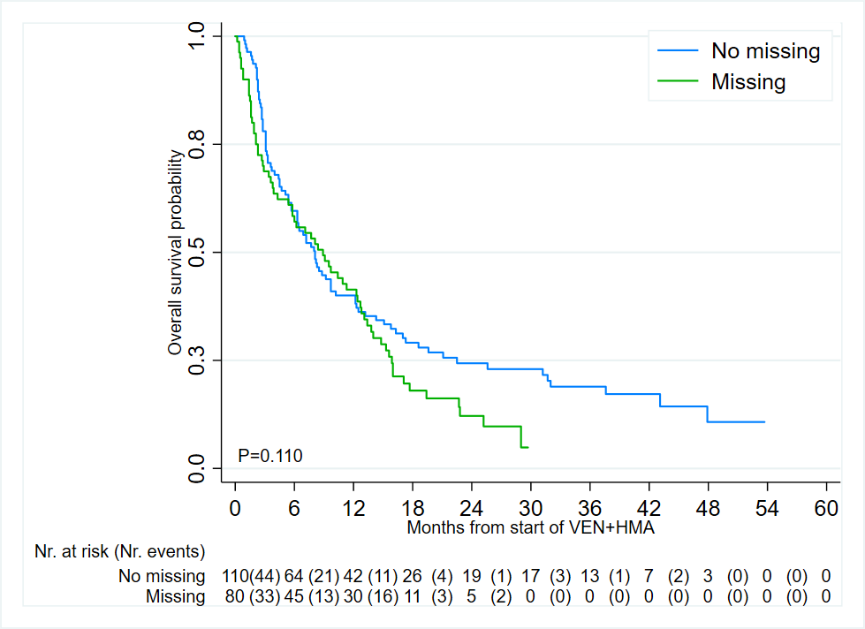


Patients not included in this analysis due to missing or inaccurate comorbidity data to compute the HCT-CI score were similar in all respect to those included, except for a higher prevalence of relapsed patients (57% vs 36%, p=0.037). Also, regarding the augmented HCT-CI score, a higher prevalence of relapsed patients was observed among those with missing data (53% vs 34%, p=0.027) as well as a slightly lower median platelets level (31 [IQR=47] vs 33 [IQR=45] p=0.050).

S3: Distribution of the factors required for the computation of the HCT-CI and augmented HCT-Ci scores (n=143)

|  | **Total** | |
| --- | --- | --- |
|  | n | (%) |
|  |  |  |
| **Arrithmia** | 5 | (3.5) |
| **Cardiovascular** | 20 | (14.0) |
| **IBD** | 0 | - |
| **Diabetes** | 16 | (11.2) |
| **Cerebrovascular disease** | 0 | - |
| **Psychiatric disturbance** | 2 | (1.4) |
| **Hepatic** |  |  |
| Mild | 13 | (9.1) |
| Moderate/severe | 2 | (1.4) |
| **Obesity** | 10 | (7.0) |
| **Infection** | 13 | (9.1) |
| **Rheumatologic** | 11 | (7.7) |
| **Peptic ulcer** | 7 | (4.9) |
| **Moderate/severe renal** | 6 | (4.2) |
| **Pulmonary** |  |  |
| Mild | 9 | (6.3) |
| Moderate | 5 | (3.5) |
| Severe | 1 | (0.7) |
| **Prior malignancy (non myeloid)** | 17 | (11.9) |
| **Heart valve disease** | 4 | (2.8) |
| **Hypoalbuminemia** | 11 | (7.7) |
| missing | 20 |  |
| **Trombocytopenia** | 83 | (58.0) |
| missing | 2 |  |
| **LDH** |  |  |
| 200 - 1000 U/L | 61 | (42.7) |
| >1000 U/L | 3 | (2.1) |
| missing | 17 |  |

HCT-CI: Hematopoietic cell transplantation comorbidity index; IBD: inflammatory bowel disease.

Percentages may not equal 100 due to rounding. Missing patients were included in HCT-CI analyses but not in augmented HCT-CI analyses.

S4: Sankey plot for SIE/SIES/GITMO criteria and HCT-CI (A) or Augmented HCT-CI (B).


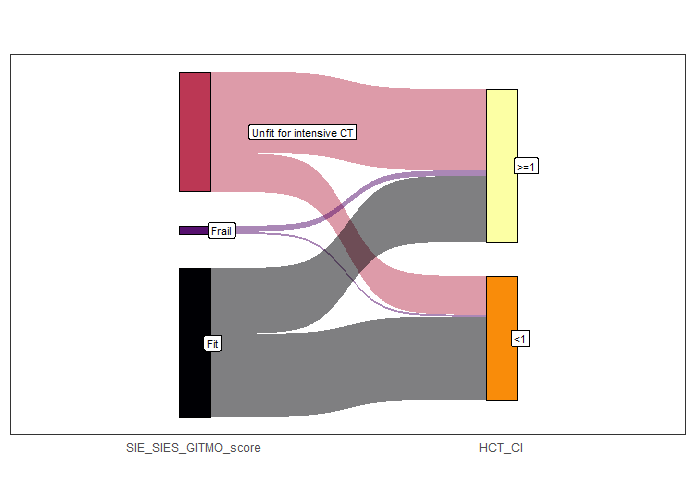


A


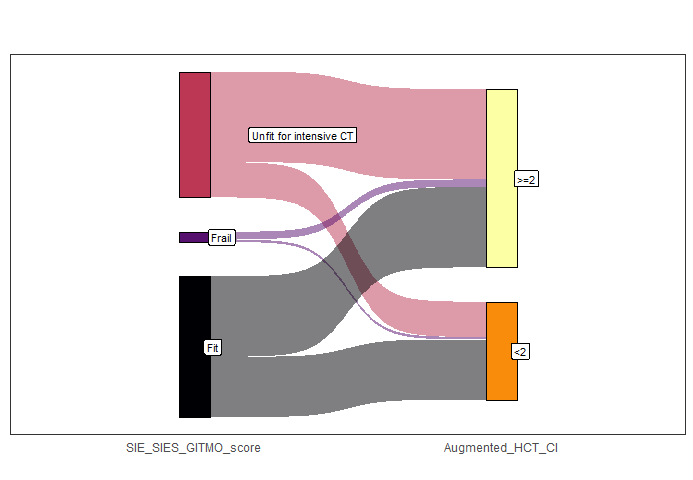


B

S5: Overall patients’ characteristics and by groups defined by the median of the scores values.

|  | **Total (n=143)** | | **HCT-CI <1**  **(n=64)** | | **HCT-CI ≥1**  **(n=79)** | |  | **Augmented HCT-CI <2 (n=39)** | | **Augmented HCT-CI ≥2 (n=71)** | |  |
| --- | --- | --- | --- | --- | --- | --- | --- | --- | --- | --- | --- | --- |
|  | n | (%) | n | (%) | n | (%) | p | n | (%) | n | (%) | p |
| **Median age at the start of the combo [IQ - IIIQ]** | 67 [54-74] | | 63.0 [50.0 – 70.5] | | 70.0 [59.0-74.0] | | 0.020 | 67.0 [53.0-72.0] | | 70 [58.0-74.0] | | 0.629 |
| Age ≤ 60 | 52 | (36.4) | 29 | (45.3) | 23 | (29.1) | 0.045 | 14 | (35.9) | 21 | (29.6) | 0.496 |
| Age > 60 | 91 | (63.6) | 35 | (54.69) | 56 | (70.9) |  | 25 | (64.1) | 50 | (70.4) |  |
| **Gender** |  |  |  |  |  |  | 0.270 |  |  |  |  | 0.892 |
| Female | 62 | (43.4) | 31 | (48.4) | 31 | (39.2) |  | 17 | (43.6) | 30 | (42.3) |  |
| Male | 81 | (56.6) | 33 | (51.6) | 48 | (60.8) |  | 22 | (56.4) | 41 | (57.8) |  |
| **AML type** |  |  |  |  |  |  | 0.009 |  |  |  |  | 0.015 |
| De novo AML | 92 | (64.3) | 49 | (76.6) | 43 | (54.4) |  | 31 | (79.5) | 37 | (52.1) |  |
| Secondary AML | 41 | (28.7) | 14 | (21.9) | 27 | (34.2) |  | 7 | (18.0) | 26 | (36.6) |  |
| Therapy related | 10 | (7.0) | 1 | (1.6) | 9 | (11.4) |  | 1 | (2.6) | 8 | (11.3) |  |
| **AML line** |  |  |  |  |  |  | 0.111 |  |  |  |  | 0.238 |
| Newly diagnosis | 36 | (25.2) | 12 | (18.8) | 24 | (30.4) |  | 8 | (20.5) | 22 | (31.0) |  |
| Relapsed or refractory | 107 | (74.8) | 52 | (81.3) | 55 | (69.6) |  | 31 | (79.5) | 49 | (69.0) |  |
| **SIE/SIES/GITMO criteria** |  |  |  |  |  |  | 0.010 |  |  |  |  | 0.272 |
| Fit | 77 | (53.9) | 43 | (67.2) | 34 | (43.0) |  | 24 | (61.5) | 32 | (45.1) |  |
| Unfit for intensive CT | 62 | (43.4) | 20 | (31.3) | 42 | (53.2) |  | 14 | (35.9) | 36 | (50.7) |  |
| Frail | 4 | (2.8) | 1 | (1.6) | 3 | (3.8) |  | 1 | (2.6) | 3 | (4.2) |  |
| **2017 ELN risk stratification by genetics**^§^ |  |  |  |  |  |  | 0.890 |  |  |  |  | 0.552 |
| Favorable | 10 | (8.1) | 4 | (7.1) | 6 | (9.0) |  | 2 | (5.9) | 6 | (10.0) |  |
| Intermediate | 67 | (54.5) | 30 | (53.6) | 37 | (55.2) |  | 21 | (61.8) | 30 | (50.0) |  |
| Adverse | 46 | (37.4) | 22 | (39.3) | 24 | (35.8) |  | 11 | (32.4) | 24 | (40.0) |  |

HCT-CI: Hematopoietic cell transplantation comorbidity index; IQ: first quartile; IIIQ: third quartile; ELN: European Leukemia Network; Percentages may not equal 100 due to rounding.

S6: Results from univariable Cox models for OS.


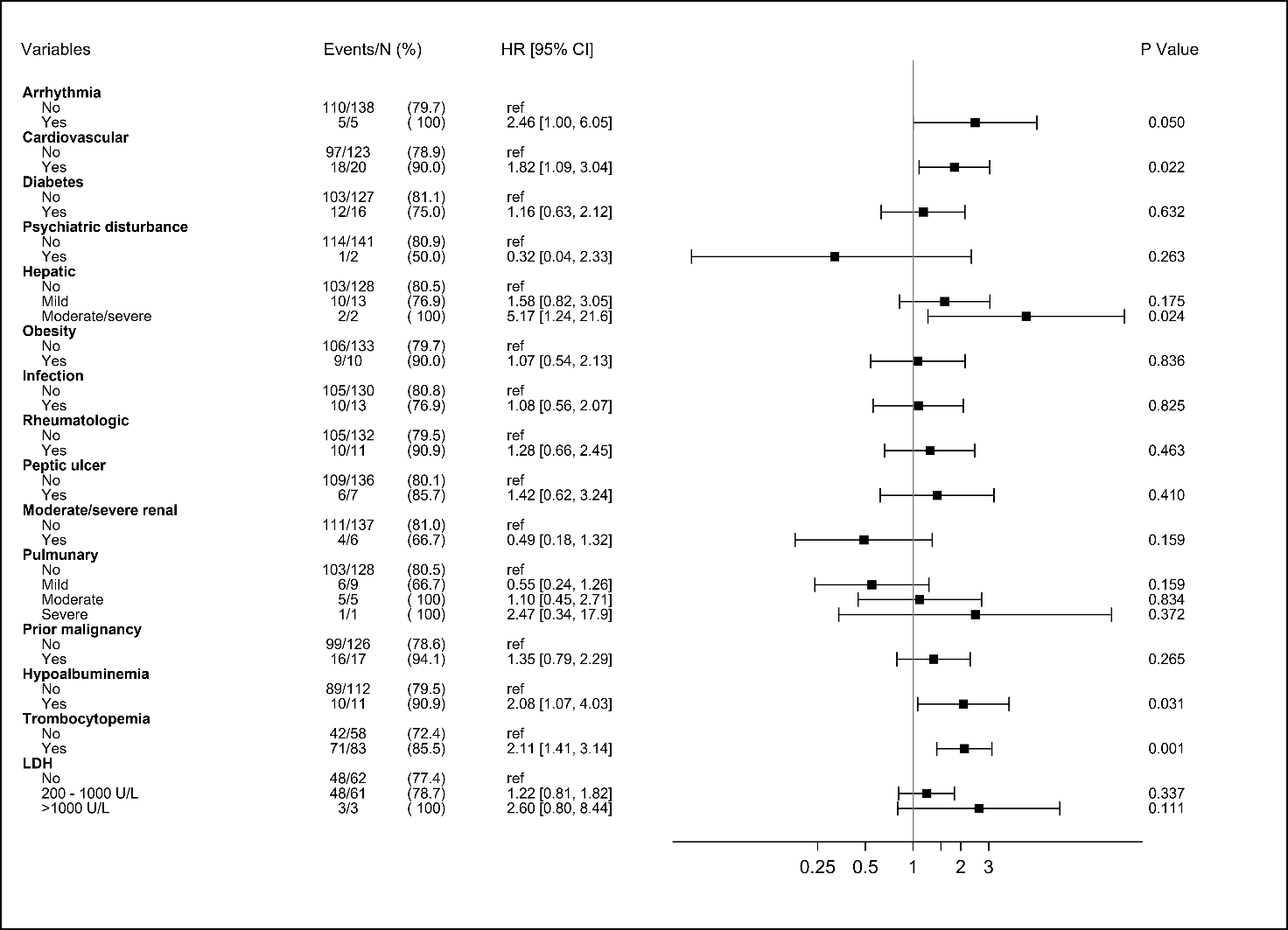
E: (death) events; HR: hazard ratio; CI: confidence interval; IBD: inflammatory bowel disease; LDH: Lactate dehydrogenase.

Multivariate Cox regression analysos confirmed Arrhythmia, Cardiovascular disease, Thrombocytopenia, and Hepatic comorbidity having an impact on OS; it should be noted that the non-significance of some comorbidities that impacted in the original creation of the scores used may be related to low number of patients affected in our set

|  | HR [95% CI] | p |
| --- | --- | --- |
| **Arrythmia** |  |  |
| No | 1 (ref) |  |
| Yes | 3.71 [1.47 – 9.37] | 0.006 |
| **Cardiovascular** |  |  |
| No | 1 (ref) |  |
| Yes | 1.68 [0.91 – 3.12] | 0.099 |
| **Thrombocytopenia** |  |  |
| No | 1 (ref) |  |
| Yes | 2.47 [1.55 – 3.95] | <0.001 |
| **Hepatic** |  |  |
| No | 1 (ref) |  |
| Mild | 2.80 [1.28 – 6.13] | 0.010 |
| Moderate/severe | 5.01 [1.16 – 21.57] | 0.030 |

S7: Kaplan-Meier curves for EFS by HCT-CI (A) and augmented HCT-CI (B) classes based on the median value

A)


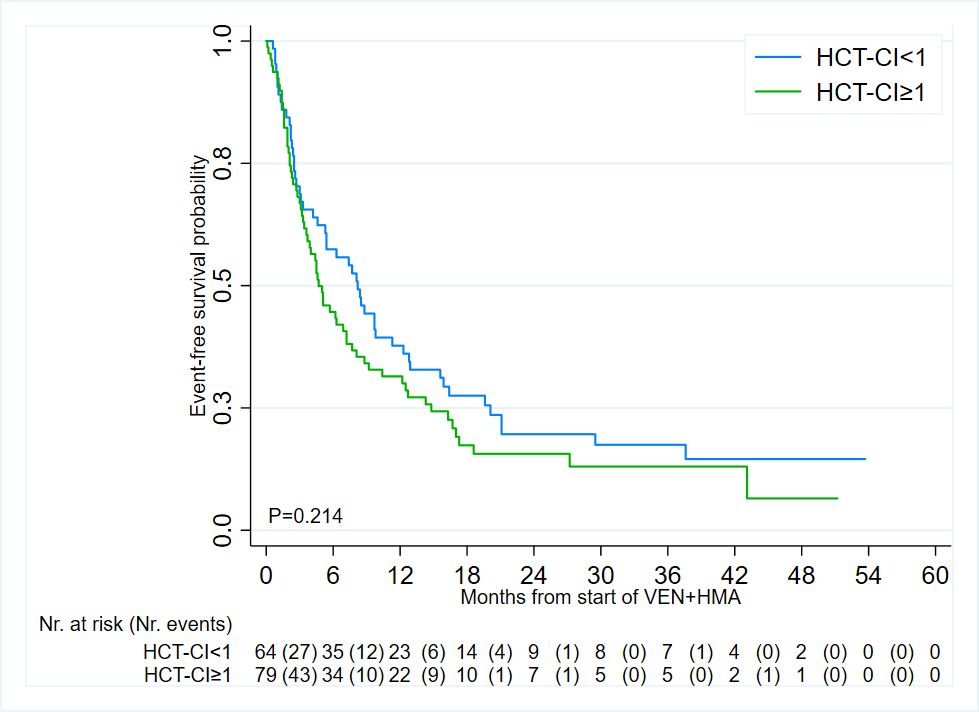


B)


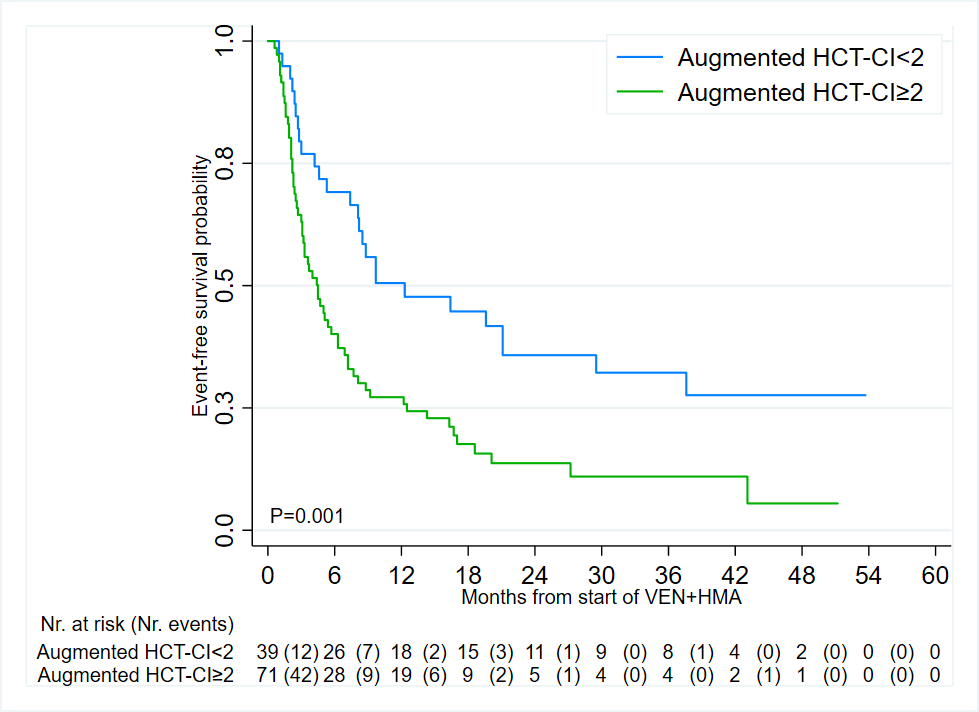


|  | Median [95% CI] |
| --- | --- |
| HCT-CI |  |
| <1 | 8.2 [4.6 – 11.3] |
| ≥1 | 4.7 [3.6 – 7.2] |
| Augmented HCT-CI |  |
| <2 | 12.3 [7.4 – 29.5] |
| ≥2 | 4.5 [3.1 – 6.3] |

S8: Multivariate Cox regression analysis of age, secondary AML, ELN risk, and augmented HCT-CI (A) and age, secondary AML, ELN risk, and augmented HCT-CI (B) impact on OS

|  | **HCT-CI** | |
| --- | --- | --- |
|  | **HR [95% CI]** | **p** |
| **HCT-CI^§^** | 1.10 [1.01 – 1.21] | 0.036 |
| **Age at start of VEN+HM†** | 1.05 [0.97 – 1.14] | 0.233 |
| **AML type** |  |  |
| De novo AML | 1 (ref) |  |
| Secondary AML or therapy related | 1.47 [0.94 – 2.29] | 0.090 |
| **2017 ELN risk stratification** |  |  |
| Favorable | 1 (ref) |  |
| Intermediate | 1.50 [0.70-3.20] | 0.296 |
| Adverse | 1.33 [0.60 – 2.95] | 0.483 |
| **AML line** |  |  |
| Newly diagnosed | 1 (ref) |  |
| Relapsed or refractory | 2.36 [1.35 – 4.11] | 0.003 |

**^§^1-unit increase; ^†^ 5-year increase**

|  | **Augmented HCT-CI** | |
| --- | --- | --- |
|  | **HR [95% CI]** | **p** |
| **Augmented HCT-CI^§^** | 1.17 [1.06 – 1.28] | 0.002 |
| **Age at start of VEN+HM†** | 1.06 [0.97 – 1.17] | 0.210 |
| **AML type** |  |  |
| De novo AML | 1 (ref) |  |
| Secondary AML or therapy related | 1.48 [0.88 – 2.49] | 0.138 |
| **2017 ELN risk stratification** |  |  |
| Favorable | 1 (ref) |  |
| Intermediate | 1.98 [0.82 – 4.77] | 0.127 |
| Frail | 1.33 [0.53 – 3.33] | 0.544 |
| **AML line** |  |  |
| Newly diagnosed | 1 (ref) |  |
| Relapsed or refractory | 2.85 [1.46 – 5.58] | 0.002 |

**^§^1-unit increase; ^†^ 5-year increase**

S9: Kaplan-Meier curves for OS by HCT-CI (A) and augmented HCT-CI (B) classes defined by Sorror 2017

A)


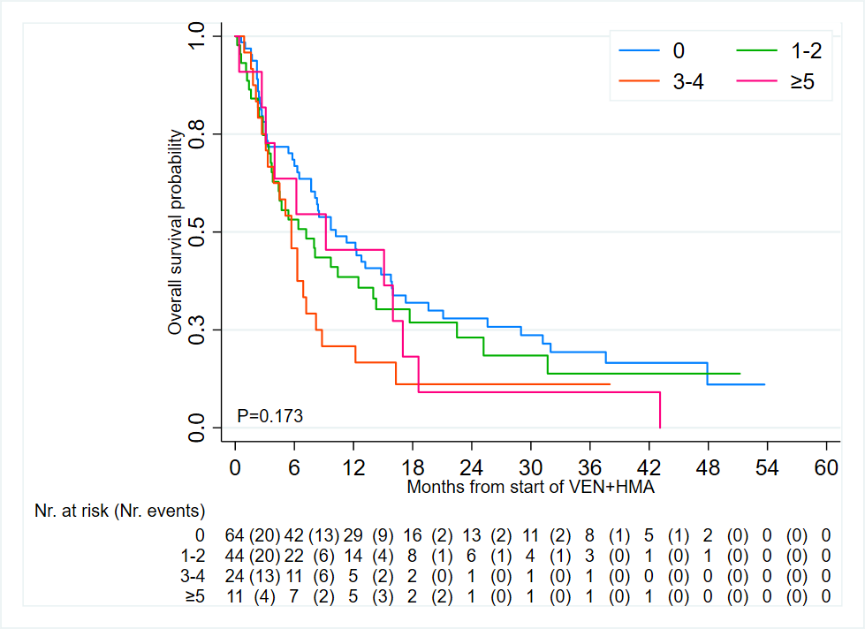


B)


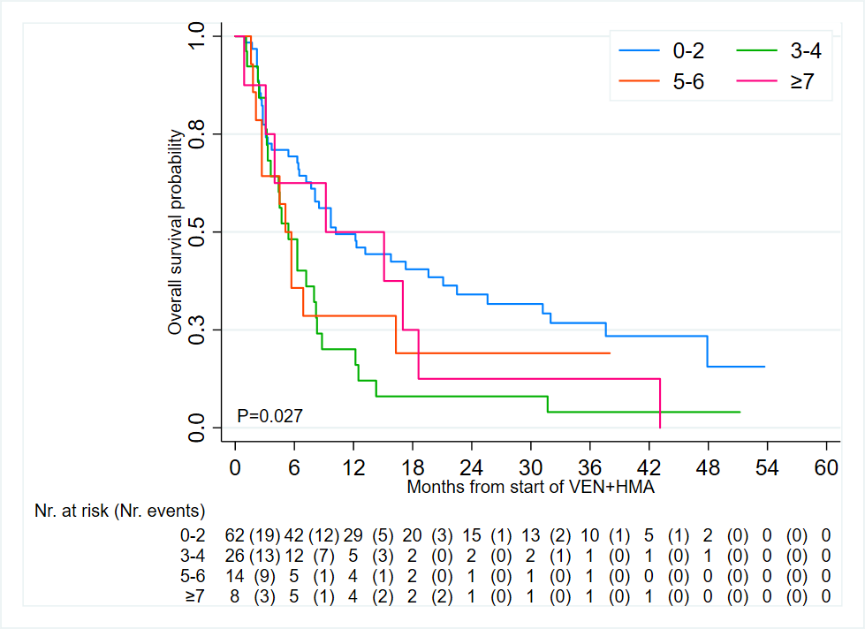


| **Sorror et al cut-offs** | **Median OS [95% CI]** |
| --- | --- |
| **HCT-CI** |  |
| 0 | 10.2 [7.7 - 15.8] |
| 1-2 | 7.2 [3.7 - 14.0] |
| 3-4 | 5.7 [3.1 – 7.2] |
| ≥5 | 9.2 [2.7 – 17.0] |
| **Augmented HCT-CI** |  |
| 0-2 | 10.2 [7.2-21.1] |
| 3-4 | 5.4 [3.3 – 8.2] |
| 5-6 | 5.1 [2.1 – 16.3] |
| ≥7 | 9.2 [0.9 – 18.6] |

S10: Kaplan-Meier curves for OS by HCT-CI (A) and augmented HCT-CI (B) classes based on the median value across young and elderly patients (age ≤60 or >60 years).


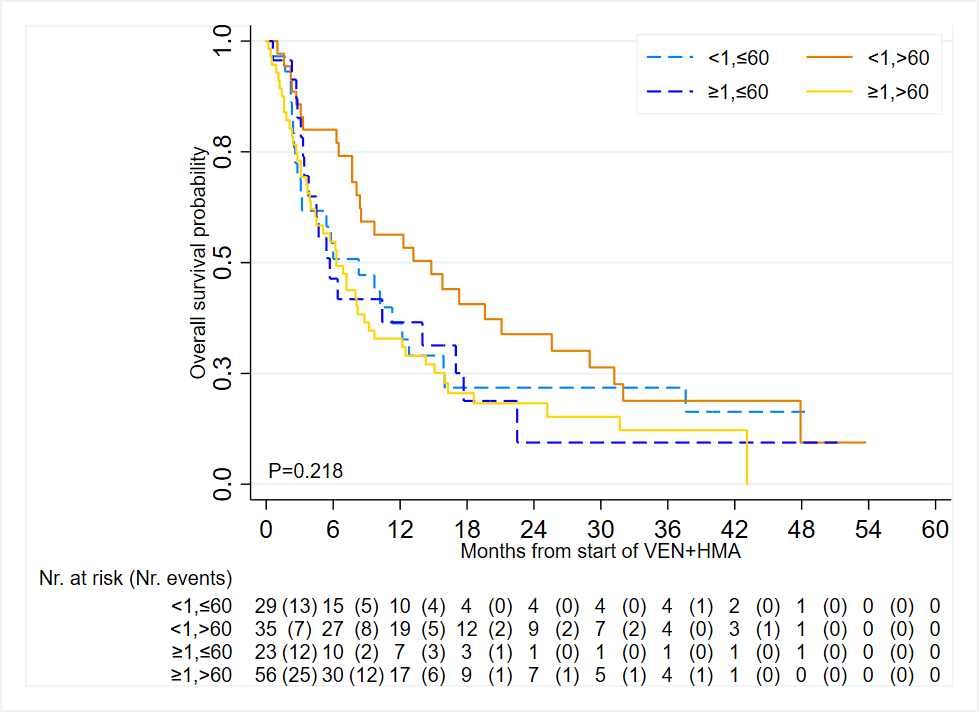


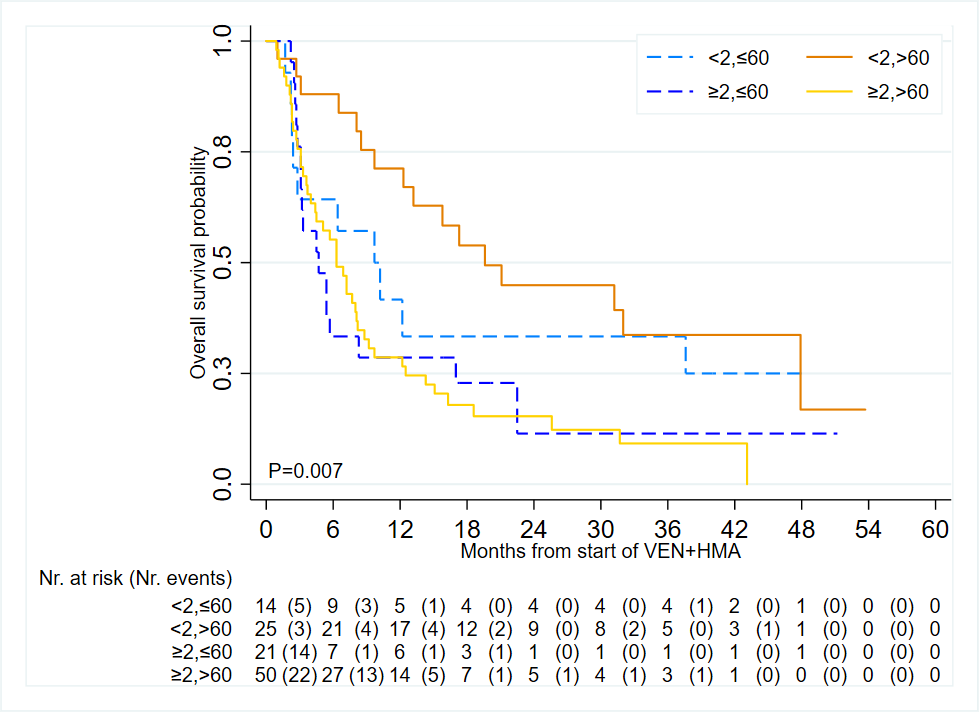


|  | **Median OS [95% CI]** |
| --- | --- |
| **HCT-CI, Age** |  |
| <1, ≤60 | 8.3 [2.8 – 12.2] |
| <1, >60 | 14.8 [7.7 – 25.6] |
| ≥1, ≤60 | 5.7 [3.4 – 17.0] |
| ≥1, >60 | 6.3 [3.9 – 8.8] |
| **Augmented HCT-CI, Age** |  |
| <2, ≤60 | 9.7 [2.3 – 37.6] |
| <2, >60 | 19.6 [9.7 – 47.9] |
| ≥2, ≤60 | 4.7 [3.1 – 8.3] |
| ≥2, >60 | 6.3 [4.0 – 8.2] |

Please note that patients with <60 years of age were enriched for relapsed or refractory AML (100%)S11: Kaplan-Meier curves for OS by HCT-CI (A) and augmented HCT-CI (B) classes based on the median value across newly diagnosed (ND) and relapse/refractory (R/R) patients.


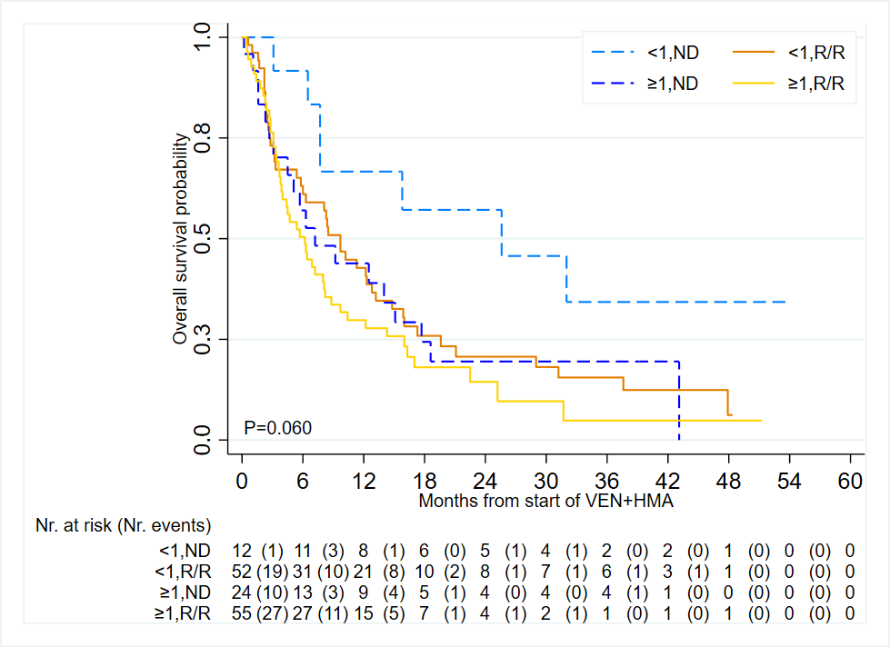


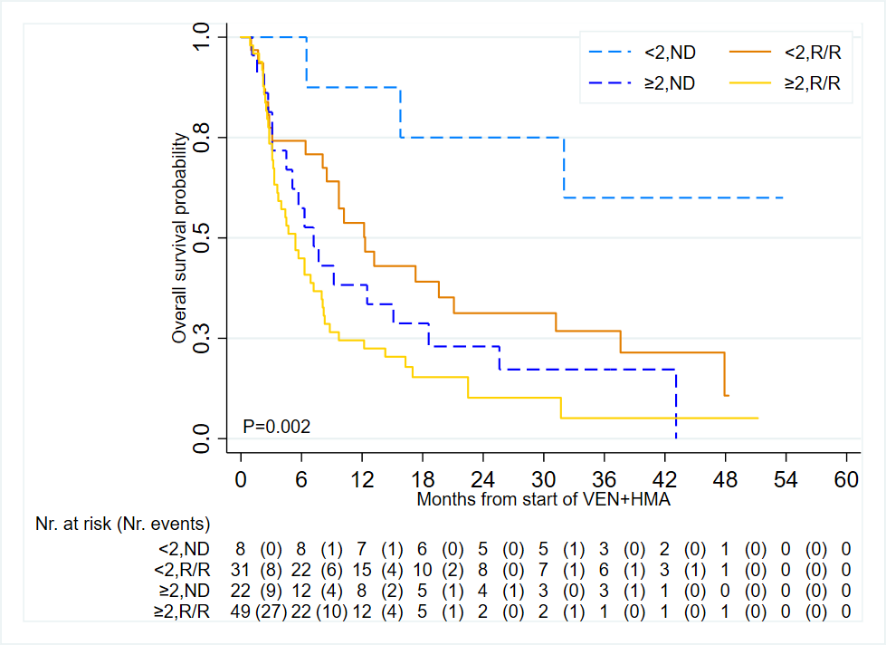


|  | **Median OS [95% CI]** |
| --- | --- |
| **HCT-CI, AML line** |  |
| <1, ND | 25.6 [6.5 – Not reached] |
| <1, R/R | 9.7 [5.8 – 12.8] |
| ≥1, ND | 7.2 [3.1 – 15.1] |
| ≥1, R/R | 6.2 [3.8 – 8.1] |
| **Augmented HCT-CI, AML line** |  |
| <2, ND | Not reached |
| <2, R/R | 12.3 [8.1 – 21.1] |
| ≥2, ND | 7.2 [3.1 – 15.1] |
| ≥2, R/R | 5.4 [3.3 – 7.2] |

S12: Distribution of the factors included in the HCT-CI and augmented HCT-CI scores by age and AML line and stratified analysis

|  | Age at start of VEN+HMA | | | | | AML line | | | | |
| --- | --- | --- | --- | --- | --- | --- | --- | --- | --- | --- |
|  | ≤60y  (n=52) | | >60y  (n=91) | | P Value | ND  (n=36) | | RE/R  (n=107) | | P Value |
|  | n | (%) | n | (%) |  | n | (%) | n | (%) |  |
| **Arrhythmia** |  |  |  |  | 0.653 |  |  |  |  | 0.102 |
| No | 51 | (98.1) | 87 | (95.6) |  | 33 | (91.7) | 105 | (98.1) |  |
| Yes | 1 | (1.9) | 4 | (4.4) |  | 3 | (8.3) | 2 | (1.9) |  |
| **Cardiovascular** |  |  |  |  | 0.001 |  |  |  |  | 0.592 |
| No | 51 | (98.1) | 72 | (79.1) |  | 30 | (83.3) | 93 | (86.9) |  |
| Yes | 1 | (1.9) | 19 | (20.9) |  | 6 | (16.7) | 14 | (13.1) |  |
| **IBD** |  |  |  |  | - |  |  |  |  | - |
| No | 52 | (100.0) | 91 | (100.0) |  | 36 | (100.0) | 107 | (100.0) |  |
| Yes | 0 |  | 0 |  |  | 0 |  | 0 |  |  |
| **Diabetes** |  |  |  |  | 0.169 |  |  |  |  | 0.228 |
| No | 49 | (94.2) | 78 | (85.7) |  | 30 | (83.3) | 97 | (90.7) |  |
| Yes | 3 | (5.8) | 13 | (14.3) |  | 6 | (16.7) | 10 | (9.4) |  |
| **Cerebrovascular disease** |  |  |  |  | - |  |  |  |  | - |
| No | 52 | (100.0) | 91 | (100.0) |  | 36 | (100.0) | 107 | (100.0) |  |
| Yes | 0 |  | 0 |  |  | 0 |  | 0 |  |  |
| **Psychiatric disturbance** |  |  |  |  | 0.534 |  |  |  |  | 0.441 |
| No | 52 | (100.0) | 89 | (97.8) |  | 35 | (97.2) | 106 | (99.1) |  |
| Yes | 0 |  | 2 | (2.2) |  | 1 | (2.8) | 1 | (0.9) |  |
| **Hepatic** |  |  |  |  | 0.789 |  |  |  |  | 0.618 |
| No | 47 | (90.4) | 81 | (89.0) |  | 32 | (88.9) | 96 | (89.7) |  |
| Mild | 5 | (9.6) | 8 | (8.8) |  | 3 | (8.3) | 10 | (9.4) |  |
| Moderate**/**severe | 0 |  | 2 | (2.2) |  | 1 | (2.8) | 1 | (0.9) |  |
| **Obesity** |  |  |  |  | 0.747 |  |  |  |  | 0.271 |
| No | 49 | (94.3) | 84 | (92.3) |  | 32 | (88.9) | 101 | (94.4) |  |
| Yes | 3 | (5.8) | 7 | (7.7) |  | 4 | (11.1) | 6 | (5.6) |  |
| **Infection** |  |  |  |  | 0.442 |  |  |  |  | 0.068 |
| No | 46 | (88.5) | 84 | (92.3) |  | 30 | (83.3) | 100 | (93.5) |  |
| Yes | 6 | (11.5) | 7 | (7.7) |  | 6 | (16.7) | 7 | (6.5) |  |
| **Rheumatologic** |  |  |  |  | 0.328 |  |  |  |  | 0.107 |
| No | 50 | (96.2) | 82 | (90.1) |  | 31 | (86.1) | 101 | (94.4) |  |
| Yes | 2 | (3.9) | 9 | (9.9) |  | 5 | (13.9) | 6 | (5.6) |  |
| **Peptic ulcer** |  |  |  |  | 0.705 |  |  |  |  | 0.368 |
| No | 49 | (94.2) | 87 | (95.6) |  | 33 | (91.7) | 103 | (96.3) |  |
| Yes | 3 | (5.8) | 4 | (4.4) |  | 3 | (8.3) | 4 | (3.7) |  |
| **Moderate/severe renal** |  |  |  |  | 0.668 |  |  |  |  | 0.642 |
| No | 49 | (94.3) | 88 | (96.7) |  | 34 | (94.4) | 103 | (92.3) |  |
| Yes | 3 | (5.8) | 3 | (3.3) |  | 2 | (5.6) | 4 | (3.7) |  |
| **Pulmonary** |  |  |  |  | 0.886 |  |  |  |  | 0.407 |
| No | 48 | (92.3) | 80 | (87.9) |  | 30 | (83.3) | 98 | (91.6) |  |
| Mild | 3 | (5.8) | 6 | (6.6) |  | 4 | (11.1) | 5 | (4.7) |  |
| Moderate | 1 | (1.9) | 4 | (4.4) |  | 2 | (5.6) | 3 | (2.8) |  |
| Severe | 0 |  | 1 | (1.1) |  | 0 |  | 1 | (0.9) |  |
| **Prior malignancy** |  |  |  |  | 0.292 |  |  |  |  | 0.668 |
| No | 48 | (92.3) | 78 | (85.7) |  | 31 | (86.1) | 95 | (88.8) |  |
| Yes | 4 | (7.7) | 13 | (14.3) |  | 5 | (13.9) | 12 | (11.2) |  |
| **Heart valve disease** |  |  |  |  | 0.297 |  |  |  |  | 0.049 |
| No | 52 | (100.0) | 87 | (95.6) |  | 33 | (91.7) | 106 | (99.1) |  |
| Yes | 0 |  | 4 | (4.4) |  | 3 | (8.3) | 1 | (0.9) |  |
| **Hypoalbuminemia** |  |  |  |  | 0.118 |  |  |  |  | 1.000 |
| No | 35 | (85.4) | 77 | (93.9) |  | 30 | (90.9) | 82 | (91.1) |  |
| Yes | 6 | (14.6) | 5 | (6.1) |  | 3 | (3) | 8 | (8.9) |  |
| missing | 11 |  | 9 |  |  | 3 |  | 17 |  |  |
| **Trombocytopenia** |  |  |  |  | 0.481 |  |  |  |  | 0.390 |
| No | 19 | (37.3) | 39 | (43.3) |  | 17 | (47.2) | 41 | (39.1) |  |
| Yes | 32 | (62.8) | 51 | (56.7) |  | 19 | (52.8) | 64 | (61.0) |  |
| missing |  |  |  |  |  |  |  |  |  |  |
| **LDH** |  |  |  |  | 0.356 |  |  |  |  | 0.342 |
| No | 23 | (52.3) | 39 | (47.6) |  | 13 | (40.6) | 49 | (52.1) |  |
| 200 - 1000 U/L | 19 | (43.2) | 42 | (51.2) |  | 19 | (59.4) | 42 | (44.7) |  |
| >1000 U/L | 2 | (4.6) | 1 | (1.2) |  | 0 |  | 3 | (3.2) |  |
| missing | 8 |  | 9 |  |  | 4 |  | 13 |  |  |
| **Median HCT-CI [IQ-IIIQ]** | 0 [0-1] | | 1 [0-3] | | 0.016 | 1.5 [0-3] | | 1 [0-2] | | 0.018 |
| **Median augmented HCT-CI [IQ-IIIQ]** | 2 [1-3] | | 2 [1-4] | | 0.495 | 3.5 [1-5] | | 2 [1-3] | | 0.031 |

ND: newly diagnosed; R/R: primary refractory or relapsed patients; HCT-CI: Hematopoietic cell transplantation comorbidity index; IQ: first-quartile; IIIQ: third quartile; HCT-CI: Hematopoietic cell transplantation comorbidity index. Percentages may not equal 100 due to rounding.

|  | Age at start of VEN+HMA | | | | AML line | | | |
| --- | --- | --- | --- | --- | --- | --- | --- | --- |
|  | ≤60y (n=52) | | >60y (n=91) | | ND (n=36) | | R/R (n=107) | |
|  | HR (95% CI) | P | HR (95% CI) | P | HR (95% CI) | P | HR (95% CI) | P |
| **Arrhythmia** |  |  |  |  |  |  |  |  |
| No | 1 (ref) |  | 1 (ref) |  | 1 (ref) |  | 1 (ref) |  |
| Yes | 1.63 (0.22-12.09) | 0.633 | 2.69 (0.97-7.43) | 0.057 | 2.63 (0.77-8.91) | 0.122 | 3.37 (0.81-13.92) | 0.094 |
| **Cardiovascular** |  |  |  |  |  |  |  |  |
| No | 1 (ref) |  | 1 (ref) |  | 1 (ref) |  | 1 (ref) |  |
| Yes | - | - | 2.38 (1.37-4.13) | 0.002 | 4.21 (1.43-12.42) | 0.009 | 1.42 (0.79-2.58) | 0.243 |
| **IBD** |  |  |  |  |  |  |  |  |
| No | 1 (ref) |  | 1 (ref) |  | 1 (ref) |  | 1 (ref) |  |
| Yes | - | - | - | - | - | - | - | - |
| **Diabetes** |  |  |  |  |  |  |  |  |
| No | 1 (ref) |  | 1 (ref) |  | 1 (ref) |  | 1 (ref) |  |
| Yes | 2.12 (0.63-7.15) | 0.227 | 1.01 (0.50-2.05) | 0.969 | 2.05 (0.67-6.27) | 0.209 | 0.96 (0.46-1.99) | 0.912 |
| **Cerebrovascular disease** |  |  |  |  |  |  |  |  |
| No | 1 (ref) |  | 1 (ref) |  | 1 (ref) |  | 1 (ref) |  |
| Yes | - | - | - | - | - | - | - | - |
| **Psychiatric disturbance** |  |  |  |  |  |  |  |  |
| No | 1 (ref) |  | 1 (ref) |  | 1 (ref) |  | 1 (ref) |  |
| Yes | - | - | 0.34 (0.05-2.42) | 0.279 | - | - | 0.84 (0.12-6.06) | 0.863 |
| **Hepatic** |  |  |  |  |  |  |  |  |
| No | 1 (ref) |  | 1 (ref) |  | 1 (ref) |  | 1 (ref) |  |
| Mild | 1.91 (0.66-5.54) | 0.232 | 1.43 (0.61-3.35) | 0.405 | 4.31 (1.14-16.36) | 0.032 | 1.22 (0.56-2.66) | 0.621 |
| Moderate/severe | - | - | 5.06 (1.18 -21.73) | 0.029 | 5.72 (0.68-48.23) | 0.109 | 9.31 (1.20-72.20) | 0.033 |
| **Obesity** |  |  |  |  |  |  |  |  |
| No | 1 (ref) |  | 1 (ref) |  | 1 (ref) |  | 1 (ref) |  |
| Yes | 1.34 (0.41-4.36) | 0. 627 | 0.96 (0.41-2.21) | 0.916 | 0.95 (0.28-3.19) | 0.936 | 1.27 (0.55-2.93) | 0.569 |
| **Infection** |  |  |  |  |  |  |  |  |
| No | 1 (ref) |  | 1 (ref) |  | 1 (ref) |  | 1 (ref) |  |
| Yes | 0.84 (0.33-2.15) | 0.715 | 1.29 (0.52-3.21) | 0.585 | 1.49 (0.55-4.02) | 0.434 | 0.98 (0.40-2.43) | 0.971 |
| **Rheumatologic** |  |  |  |  |  |  |  |  |
| No | 1 (ref) |  | 1 (ref) |  | 1 (ref) |  | 1 (ref) |  |
| Yes | 0.31 (0.04-2.29) | 0.252 | 1.96 (0.97-3.98) | 0.062 | 2.83 (1.03-7.75) | 0.043 | 0.87 (0.35-2.15) | 0.758 |
| **Peptic ulcer** |  |  |  |  |  |  |  |  |
| No | 1 (ref) |  | 1 (ref) |  | 1 (ref) |  | 1 (ref) |  |
| Yes | 0.61 (0.15-2.54) | 0.496 | 3.33 (1.16-9.56) | 0.025 | 4.93 (1.29-18.90) | 0.020 | 0.82 (0.26-2.60) | 0.735 |
| **Moderate/severe renal** |  |  |  |  |  |  |  |  |
| No | 1 (ref) |  | 1 (ref) |  | 1 (ref) |  | 1 (ref) |  |
| Yes | 0.27 (0.04-1.99) | 0.199 | 1.14 (0.36-3635) | 0.827 | 1.29 (0.30-5.53) | 0.729 | 0.29 (0.07-1.17) | 0.083 |
| **Pulmonary** |  |  |  |  |  |  |  |  |
| No | 1 (ref) |  | 1 (ref) |  | 1 (ref) |  | 1 (ref) |  |
| Mild | 0.35 (0.08-1.49) | 0.156 | 0.76 (0.28-2.08) | 0.590 | 1.51 (0.45-5.13) | 0.507 | 0.32 (0.10-1.04) | 0.058 |
| Moderate | 0.49 (0.07-3.64) | 0.483 | 1.48 (0.54-4. 10) | 0.448 | 2.53 (0.57-11.21) | 0.221 | 0.76 (0.24-2.40) | 0.635 |
| Severe | - | - | 2.80 (0.38-20.70) | 0.312 | - |  | 2.14 (0.29-15.57) | 0.454 |
| **Prior malignancy** |  |  |  |  |  |  |  |  |
| No | 1 (ref) |  | 1 (ref) |  | 1 (ref) |  | 1 (ref) |  |
| Yes | 2.87 (0.97-8.49) | 0.057 | 1.17 (0.63-2.18) | 0.620 | 0.69 (0.23-2.03) | 0.500 | 2.29 (1.23-4.27) | 0.009 |
| **Heart valve disease** |  |  |  |  |  |  |  |  |
| No | 1 (ref) |  | 1 (ref) |  | 1 (ref) |  | 1 (ref) |  |
| Yes | - | - | 0.97 (0.35-2.69) | 0.953 | 0.89 (0.26-3.08) | 0.858 | - | - |
| **Hypoalbuminemia** |  |  |  |  |  |  |  |  |
| No | 1 (ref) |  | 1 (ref) |  | 1 (ref) |  | 1 (ref) |  |
| Yes | 1.12 (0.46-3.20) | 0.692 | 4.04 (1.57-10.43) | 0.004 | 1.99 (0.57-6.90) | 0.277 | 2.19 (0.99-4.84) | 0.053 |
| **Trombocytopenia** |  |  |  |  |  |  |  |  |
| No | 1 (ref) |  | 1 (ref) |  | 1 (ref) |  | 1 (ref) |  |
| Yes | 2.58 (1.20-5.54) | 0.029 | 1.90 (1.17-3.08) | 0.009 | 2.68 (1.17-6.18) | 0.020 | 1.87 (1.19-2.95) | 0.007 |
| **LDH** |  |  |  |  |  |  |  |  |
| No | 1 (ref) |  | 1 (ref) |  | 1 (ref) |  | 1 (ref) |  |
| 200 - 1000 U/L | 1.05 (0.52-2.14) | 0.893 | 1.42 (0.87-2.32) | 0.165 | 1.11 (0.46-2.63) | 0.821 | 1.33 (0.84-2.10) | 0.225 |
| >1000 U/L | 1.26 (0.29-5.54) | 0.760 | 97.85 (6.02-1589.27) | 001 | - |  | 2.50 (0.76-8.19) | 0.130 |
| **HCT-CI** |  |  |  |  |  |  |  |  |
| <1 | 1 (ref) |  | 1 (ref) |  | 1 (ref) |  | 1 (ref) |  |
| ≥1 | 0.99 (0.53 – 1.85) | 0.981 | 1.72 (1.06 – 2.79) | 0.027 | 2.23 (0.94-5.35) | 0.070 | 1.29 (0.85-1.97) | 0.232 |
| **Augmented HCT-CI** |  |  |  |  |  |  |  |  |
| <2 | 1 (ref) |  | 1 (ref) |  | 1 (ref) |  | 1 (ref) |  |
| ≥2 | 1.40 (0.63 – 3.11) | 0.412 | 2.83 (1.56 – 5.15) | 0.001 | 4.49 (1.30-15.46) | 0.017 | 1.93 (1.15-3.25) | 0.013 |

HR: hazard ratio; CI: confidence interval; IBD: inflammatory bowel disease; LDH: Lactate dehydrogenase; ND: newly diagnosed; R/R: primary refractory or relapsed patients; HCT-CI: Heamopoietic cell transplantation comorbidity index

S13: AVALON Cooperative Group

| **Contributor** | **Affiliation** |
| --- | --- |
| Adriano Venditti | U.O.C. Ematologia, A.O.U. Fond. Policlinico Tor Vergata |
| Agostino Tafuri | U.O.C. Ematologia, A.O.U. Sant'Andrea, Roma |
| Alessandra Romano | A.O.U. Policlinico Vittorio Emanuele, Catania |
| Alessandro Cignetti | Divisione Universitaria di Ematologia e Terapie Cellulari, A.O. Ordine Mauriziano, Torino |
| Alfredo Molteni | U.O.C. Ematologia – CTMO, ASST Cremona, Cremona |
| Annalisa Imovilli | Dip. Oncologico e Tecnologie avanzate, IRCCS Arcispedale S. Maria Nuova, Reggio Emilia |
| Bianca Serio | A.O.U. S. Giovanni di Dio e Ruggi d'Aragona, Università di Salerno, Salerno |
| Bruna Messere | Dip. Oncopneumoematologico, A.O.R.N. “A. Cardarelli”, Napoli |
| Calogero Vetro | A.O.U. Policlinico Vittorio Emanuele, Catania |
| Carla Mazzone | Dep. Hematology S. Eugenio Ospital, Roma |
| Carmine Selleri | A.O.U. S. Giovanni di Dio e Ruggi d'Aragona, Università di Salerno, Salerno |
| Chiara Zingaretti | IRCCS Istituto Romagnolo per lo studio dei Tumori “Dino Amadori” - IRST S.r.l., Meldola |
| Claudia Basilico | ASST-Settelaghi, Ospedale di Circolo-Fondazione Macchi, Varese |
| Claudio Cerchione | IRCCS Istituto Romagnolo per lo studio dei Tumori “Dino Amadori” - IRST S.r.l., Meldola |
| Corrado Tarella | Div. Oncoematologia, European Institute of Oncology, Milano |
| Cristina Papayannidis | Dip. di Medicina Specialistica, Diagnostica e Sperimentale, Università di Bologna, Bologna |
| Daniela Cilloni | Department of Clinical and Biological Sciences, University of Turin |
| Daniele Mattei | S. C. Ematologia, A.S.O. S. Croce e Carle, Cuneo |
| Davide Griguolo | S. C. Ematologia, A.O.U. Giuliano Isontina, Trieste |
| Elisa Roncoroni | Department of Hematology Oncology, Fondazione IRCCS Policlinico San Matteo, Pavia |
| Elisabetta Abruzzese | Dep. Hematology S. Eugenio Ospital, Roma |
| Elisabetta Petracci | IRCCS Istituto Romagnolo per lo studio dei Tumori “Dino Amadori” - IRST S.r.l., Meldola |
| Elisabetta Todisco | Div. Oncoematologia, European Institute of Oncology, Milano |
| Endri Mauro | Azienda U.L.S.S.9 Ospedale Regionale Cà Foncello, Treviso |
| Erika Borlenghi | Department of Hematology, ASST Spedali Civili di Brescia, Brescia |
| Ernesta Audisio | SC Ematologia 2, Dip. di Ematologia e Oncologia, A.O.U. Città della Salute e della Scienza, Torino |
| Fabio Ciceri | U.O. Ematologia e TMO, Ospedale S.Raffaele, Milano |
| Federica Gigli | Div. Oncoematologia, European Institute of Oncology, Milano |
| Federica Monaco | AUSL della Romagna, Ospedale S. Maria delle Croci, Ravenna |
| Federico Lussana | Department of Oncology and Hematology University of Milan, and Azienda SocioSanitaria Territoriale Papa Giovanni XXIII, Bergamo |
| Felicetto Ferrara | Dip. Oncopneumoematologico, A.O.R.N. “A. Cardarelli”, Napoli |
| Flavia Rivellini | Osp. “A. Tortora” di Pagani, Pagani |
| Francesco Di Raimondo | A.O.U. Policlinico Vittorio Emanuele, Catania |
| Francesco Lanza | AUSL della Romagna, Ospedale S. Maria delle Croci, Ravenna |
| Francesco Zaja | S. C. Ematologia, A.O.U. Giuliano Isontina, Trieste |
| Giorgio Priolo | SC Ematologia 2, Dip.di Ematologia e Oncologia, AOU Città della Salute e della Scienza, Torino |
| Giovanni Marconi | IRCCS Istituto Romagnolo per lo studio dei Tumori “Dino Amadori” - IRST S.r.l., Meldola |
| Giovanni Martinelli | IRCCS Istituto Romagnolo per lo studio dei Tumori “Dino Amadori”, IRST S.r.l., Meldola |
| Giuliana Rizzuto | Department of Oncology and Hematology University of Milan, and Azienda SocioSanitaria Territoriale Papa Giovanni XXIII, Bergamo |
| Giuseppe Rossi | Department of Hematology, ASST Spedali Civili di Brescia, Brescia |
| Serena Luponio | A.O.U. S. Giovanni di Dio e Ruggi d'Aragona, Università di Salerno, Salerno |
| Ilenia Manfra | A.O.R.N. “S. Giuseppe Moscati”, Avellino |
| Irene Urbino | SC Ematologia 2, Dipartmento di Ematologia e Oncologia, AOU Città della Salute e della Scienza, Torino |
| Irene Valli | IRCCS Istituto Romagnolo per lo studio dei Tumori “Dino Amadori” - IRST S.r.l., Meldola |
| Jacopo Nanni | Dip. di Medicina Specialistica, Diagnostica e Sperimentale, Università di Bologna, Bologna |
| Katia Codeluppi | Dip. Oncologico e Tecnologie avanzate, IRCCS Arcispedale S. Maria Nuova, Reggio Emilia |
| Liliana Calabrese | Div. Oncoematologia, European Institute of Oncology, Milano |
| Luana Fianchi | Istituto di Ematologia, Fondazione Policlinico Universitario “A. Gemelli” IRCCS, Roma |
| Luca Facchini | Dip. Oncologico e Tecnologie avanzate, IRCCS Arcispedale S. Maria Nuova, Reggio Emilia |
| Luca Maurillo | U.O.C. Ematologia, A.O.U. Fond. Policlinico Tor Vergata |
| Maria Paola Martelli | Department of Medicine and Surgery, Perugia University, "Santa Maria della Misericordia" Hospital, Perugia |
| Maria Benedetta Giannini | IRCCS Istituto Romagnolo per lo studio dei Tumori “Dino Amadori” - IRST S.r.l., Meldola |
| Maria Chiara Abbenante | Dip. Onco-ematologia IRCCS Casa Sollievo della Sofferenza, S.Giovanni Rotondo (FG) |
| Mariarita Sciumè | U.O.C. Oncoematologia, Fond. IRCCS Ca' Granda Ospedale Maggiore Policlinico di Milano, Milano |
| Michele Gottardi | Azienda U.L.S.S.9 Ospedale Regionale Cà Foncello, Treviso |
| Michelina Dargenio | Hematology and SCT Unit, “Vito Fazzi” Hospital, Lecce |
| Monia Lunghi | Divisione di Ematologia, Dip.di Medicina Traslazionale, Università del Piemonte Orientale, Novara |
| Monica Fumagalli | Divisione di Ematologia Osp. S. Gerardo ASST Monza, Monza |
| Nicola Cascavilla | Dip. Onco-ematologia IRCCS Casa Sollievo della Sofferenza, S.Giovanni Rotondo (FG) |
| Nicola Di Renzo | Hematology and SCT Unit, “Vito Fazzi” Hospital, Lecce |
| Nicola Stefano Fracchiolla | U.O.C. Oncoematologia, Fond. IRCCS Ca' Granda Ospedale Maggiore Policlinico di Milano, Milano |
| Paolo De Fabritis | Dep. Hematology S. Eugenio Ospital, Roma |
| Pasquale De Roberto | U.O.C. Oncoematologia, Fond. IRCCS Ca' Granda Ospedale Maggiore Policlinico di Milano, Milano |
| Patrizia Zappasodi | Department of Hematology Oncology, Fondazione IRCCS Policlinico San Matteo, Pavia |
| Prassede Salutari | Dipartimento di Ematologia e Madicina Trasfusionale – Ospedale Civile di Pescara, Pescara |
| Raffaele Palmieri | U.O.C. Ematologia, A.O.U. Fond. Policlinico Tor Vergata |
| AnnaMaria Della Corte | A.O.U. S. Giovanni di Dio e Ruggi d'Aragona, Università di Salerno, Salerno |
| Roberta Volpi | IRCCS Istituto Romagnolo per lo studio dei Tumori “Dino Amadori” - IRST S.r.l., Meldola |
| Roberto Cairoli | SC Ematologia, ASST Grande Ospedale Metropolitano Niguarda, Milano |
| Simona Menna | Div. Oncoematologia, European Institute of Oncology, Milano |
| Sofia Sciabolacci | Department of Medicine – Section of Hematology and Clinical Immunology, Perugia University, "Santa Maria della Misericordia" Hospital, Perugia |
| Susanna Gallo | Divisione Universitaria di Ematologia e Terapie Cellulari, A.O. Ordine Mauriziano, Torino |
| Valentina Oliva | Dip. Oncopneumoematologico, A.O.R.N. “A. Cardarelli”, Napoli |
| Valeria Cardinali | Department of Medicine and Surgery, Perugia University, "Santa Maria della Misericordia" Hospital, Perugia |
| Vincenza Martini | U.O.C. Ematologia, Osp. F. Spaziani, Frosinone |
| Vincenzo Federico | Hematology and SCT Unit, “Vito Fazzi” Hospital, Lecce |
| Viviana Amato | Div. Oncoematologia, European Institute of Oncology, Milano |

S-AI: DALL.E 3.0 Input for graphical abstract picture on Dec 26^th^ 2023.

create a visual representation of patients of the same age with varying comorbidities and their outcomes after therapy. Here's a detailed description for the image:

The scene is divided into two halves.

On the left half, we see a patient with no comorbidities. This patient is depicted sitting up in a hospital bed, looking healthy and smiling. There's a chart at the foot of the bed showing positive progress. The background includes a bright window with a view of a sunny day, symbolizing recovery and good health.

On the right half, there's a patient with multiple comorbidities. This patient appears more fatigued and is lying down, with several medical monitors around the bed displaying various readings. The background on this side is more subdued, with a window showing a cloudy day, symbolizing a more challenging recovery process.

Both patients are of different descents. The patient without comorbidities is Middle-Eastern, and the one with comorbidities is Hispanic. Both are depicted with neutral expressions to maintain a respectful representation of their health conditions. Both patients have approximately the same age.

This visual aims to convey the contrast in health outcomes after therapy in patients with varying health backgrounds, without stigmatizing or being insensitive to either group.
